# Global metabolite profiling of mice with high-fat diet-induced obesity chronically treated with AMPK activators R118 or metformin reveals tissue-selective alterations in metabolic pathways

**DOI:** 10.1186/1756-0500-7-674

**Published:** 2014-09-25

**Authors:** Yonchu Jenkins, Tian-Qiang Sun, Yingwu Li, Vadim Markovtsov, Gerald Uy, Lisa Gross, Dane A Goff, Simon J Shaw, Luke Boralsky, Rajinder Singh, Donald G Payan, Yasumichi Hitoshi

**Affiliations:** Rigel Pharmaceuticals, Inc, South San Francisco, CA USA

**Keywords:** AMPK, Metformin, Complex I, Mitochondria, Diabetes, Metabolomics, Skeletal muscle, Metabolism, Small molecule, Diet-induced obese mice

## Abstract

**Background:**

The novel small molecule R118 and the biguanide metformin, a first-line therapy for type 2 diabetes (T2D), both activate the critical cellular energy sensor 5′-AMP-activated protein kinase (AMPK) via modulation of mitochondrial complex I activity. Activation of AMPK results in both acute responses and chronic adaptations, which serve to restore energy homeostasis. Metformin is thought to elicit its beneficial effects on maintenance of glucose homeostasis primarily though impacting glucose and fat metabolism in the liver. Given the commonalities in their mechanisms of action and that R118 also improves glucose homeostasis in a murine model of T2D, the effects of both R118 and metformin on metabolic pathways *in vivo* were compared in order to determine whether R118 elicits its beneficial effects through similar mechanisms.

**Results:**

Global metabolite profiling of tissues and plasma from mice with diet-induced obesity chronically treated with either R118 or metformin revealed tissue-selective effects of each compound. Whereas metformin treatment resulted in stronger reductions in glucose and lipid metabolites in the liver compared to R118, upregulation of skeletal muscle glycolysis and lipolysis was apparent only in skeletal muscle from R118-treated animals. Both compounds increased β-hydroxybutyrate levels, but this effect was lost after compound washout. Metformin, but not R118, increased plasma levels of metabolites involved in purine metabolism.

**Conclusions:**

R118 treatment but not metformin resulted in increased glycolysis and lipolysis in skeletal muscle. In contrast, metformin had a greater impact than R118 on glucose and fat metabolism in liver tissue.

**Electronic supplementary material:**

The online version of this article (doi:10.1186/1756-0500-7-674) contains supplementary material, which is available to authorized users.

## Background

The widely used type 2 diabetes therapy metformin and the recently reported investigational drug R118 (international application publication no. WO 2012/016217, February 2, 2012) are both mitochondrial complex I inhibitors [1–3] that mediate their beneficial effects on glucose homeostasis partly through modulation of the 5′-adenosine monosphate-activated protein kinase (AMPK), a critical sensor of cellular energy status. R118 is a mechanistic analog of the well-characterized small molecule R419 [4] and activates AMPK by inhibiting mitochondrial complex I with a potency of ~70 nM, comparable to the reported R419 potency of 100 nM. Chronic R118 treatment was recently shown to improve exercise performance in aged, obese mice that display many of the functional and molecular characteristics of peripheral arterial disease [1]. Metformin also activates AMPK via complex I inhibition, although the potency displayed against complex I, and consequent AMPK activation, is substantially weaker (mM) compared to R118. However, glucose homeostasis is maintained by metformin via both AMPK-dependent and independent mechanisms, with the liver thought to be the primary site of metformin action [5–10]. The mechanistic bases of metformin’s therapeutic benefits are still being debated despite extensive characterization of its effects both *in vitro* and *in vivo*. Suppression of hepatic glucose production by metformin has been attributed to a block in glucagon-stimulated glucose synthesis, which is mediated by a decrease in adenylate cyclase activity resulting from the inhibition of complex I [11]. Metformin was also recently shown to inhibit mitochondrial glycerophosphate dehydrogenase (mGPD), which suppresses glucose production from lactate and glycerol precursors [12]. In addition, chronic metformin treatment also decreased liver lipid accumulation by AMPK-mediated inhibition of acetyl-CoA carboxylase activity (ACC), which also contributes to increased insulin sensitivity [13].

Here, we extend and complement the existing studies by analyzing the chronic effects on metabolic pathways in mice with diet-induced obesity (DIO) treated with R118 or metformin. We performed global metabolite profiling on tissues and plasma from R118-treated DIO mice to better understand the effects of chronic administration of the class of compounds represented by R118 on nutrient metabolism *in vivo*. Further, the activity of R118 was directly compared to that of metformin. Given the similarities in their molecular mechanisms of action, we sought to identify both the common and the distinguishing features between R118 and metformin on biochemical pathways in liver, skeletal muscle, and adipose tissue.

## Methods

### Animal care and treatment

Male C57BL/6 J DIO mice fed with a high-fat diet (HFD, 60% of kcal from fat) since 6 weeks of age were purchased from Jackson Laboratories (Bar Harbor, ME). DIO mice were acclimated at Rigel for 4 weeks prior to study initiation. Mice were housed 4 per cage in a room with a 12-12 hour light/dark cycle and were given HFD (D12492, Research Diets, Inc., New Brunswick, NJ) and drinking water *ad libitum*. Rigel’s Institutional Animal Care and Use Committee approved all procedures (IACUC Protocol No. Rigel 2-2012).

### Metabolomics study

DIO mice (21 weeks of age, 17 weeks on HFD) were weighed and randomized into study groups so that each group had similar average body weight. Mice were treated with either HFD (n = 12), HFD formulated with 200 mg/kg R118 (n = 12), or HFD formulated with 5 g/kg metformin (n = 12). Dose selection for R118 was determined by identifying the compound level required to yield an average 24 hour exposure similar to that required for improvement of oral glucose tolerance by R118 in *db/db* leptin-receptor deficient mice (average Area Under the Curve (AUC)_0-24_ of approximately 2300 ng•hr/mL, data not shown). Dose titration for metformin was performed to determine the minimum dose required for improvement in oral glucose tolerance without accompanying body weight reduction (data not shown). At 4 weeks of compound treatment, an oral glucose tolerance test (OGTT) was performed. Glucose was administered orally at 2 g/kg following a 6-hour fast with food removal at 5 AM. Tail vein bleeds were used to measure blood glucose at 0, 30, 60, 90, and 120 minutes after glucose challenge (Breeze2, Bayer). At 5 weeks of treatment, 6 mice/treatment group were euthanized by CO_2_ asphyxiation followed by cardiac puncture at 6 AM. Liver, gastrocnemius muscle, visceral adipose tissue, and plasma were collected from each animal. Solid tissues were submerged in liquid nitrogen immediately after collection. For the remaining 6 mice in each treatment group, compound-formulated HFD was replaced with control HFD at 6 AM, followed by liver, gastrocnemius muscle, white adipose tissue, and plasma collection as above 24 hours after the chow replacement. All samples were stored at -80°C prior to shipment to Metabolon for unbiased metabolite analysis (Durham, NC) [14–16]. Biochemical data were analyzed using Welch’s two-sample t-tests.

## Results

### R118 has a stronger chronic effect on skeletal muscle metabolism compared to liver and adipose tissue

DIO mice were treated using high fat diet formulated with either R118 (200 mg/kg HFD) or metformin (5 g/kg HFD). An OGTT test was performed after 4 weeks of treatment to confirm the biologic activity of both compounds (Additional file 1: Figure S1) prior to sample collection. Oral glucose tolerance was improved without significant effects on body weight at the doses utilized for both compounds, increasing the likelihood that any observed changes in biochemical pathways will be a direct result of compound treatment, rather than indirectly due to body weight alterations.

After functional confirmation of compound activity, samples from the treated animals were collected under two different conditions for metabolite profiling. Tissues from half the animals in each treatment group were harvested at 6 AM, towards the end of the 12 hour active cycle when plasma compound concentrations are at maximal levels. In order to determine the chronic effects on metabolic pathways *in vivo* for both R118 and metformin, compound-formulated HFD was replaced with control HFD at 6 AM for the remaining animals in each treatment group to wash out the drugs, with sample collection 24 hours after chow replacement. Drug levels were measured in the plasma samples from the two harvest conditions to confirm that compound concentrations were reduced in the washout samples (Additional file 2: Figure S2). Metformin plasma concentrations in mice harvested at maximal compound levels were about 6.1 μg/mL, about 4-fold higher than plasma concentrations reported after single administration of an 850 mg metformin dose to T2D patients [17]. In the drug washout samples, plasma metformin concentrations dropped to about 0.05 μg/mL whereas R118 plasma concentrations were below the limit of quantitation.

**Figure 1:**
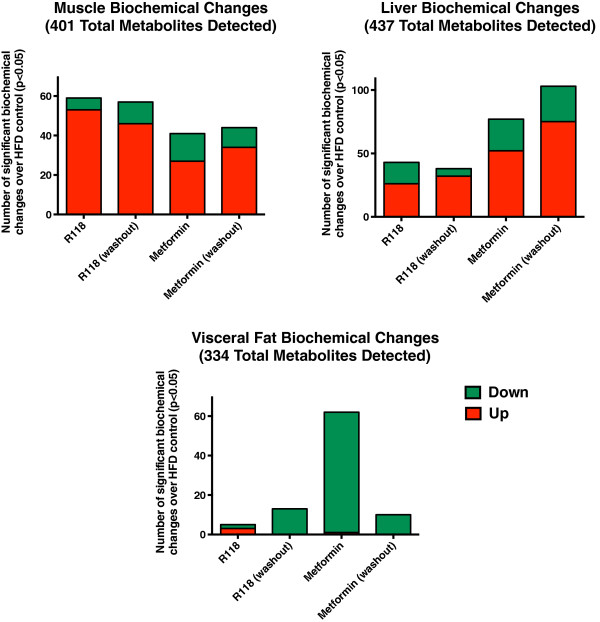
**Biochemical changes detected in skeletal muscle, liver, and adipose tissue after 5 week treatment with either R118 or metformin.** Male HFD DIO C57BL/6 mice (21 weeks old, 17 weeks on HFD) were fed using control HFD, HFD formulated with 200 mg/kg R118, or HFD formulated with 5 g/kg metformin (n = 12/group). After five weeks of treatment, liver, skeletal muscle, adipose tissue, and plasma were collected for half of the mice in each group. For the remaining mice, compound-formulated HFD was replaced with control HFD for 24 hours (washout), followed by similar sample collection. For each tissue, the number of metabolites where the difference between drug-treated versus control HFD has p < 0.05 is shown in the graphs. Further breakdown of the significant changes into metabolites that are increased versus decreased compared to the control HFD is indicated by the color coding, where green is decreased with drug treatment relative to HFD control and red is increased with drug treatment relative to control HFD.

Comparison across skeletal muscle, liver and adipose tissue of the total number of metabolites that were significantly altered (P < 0.05) between each treatment versus the HFD control shows differential responses to R118 and metformin for each tissue profiled (Figure 1). In skeletal muscle, more metabolites were altered relative to HFD control for R118-treated animals compared to metformin. In contrast, metformin treatment resulted in more liver and adipose changes than R118. Under conditions of drug washout, a similar number of biochemical differences are maintained in skeletal muscle from R118-treated mice, demonstrating that chronic R118 dosing leads to changes in skeletal muscle metabolism that are sustained in the absence of drug. Metformin effects on hepatic metabolism actually become more pronounced in the washout samples, suggesting that the immediate effects of compound treatment may obscure the detection of chronic changes in biochemical pathways. Although 18.5% of the total metabolites detected in adipose tissue were significantly affected by metformin treatment, this percentage dropped to 3% in the absence of drug, indicating that metformin effects on adipose tissue are not sustained.

### R118 affects both glucose and fat metabolism in skeletal muscle

**Figure 2:**
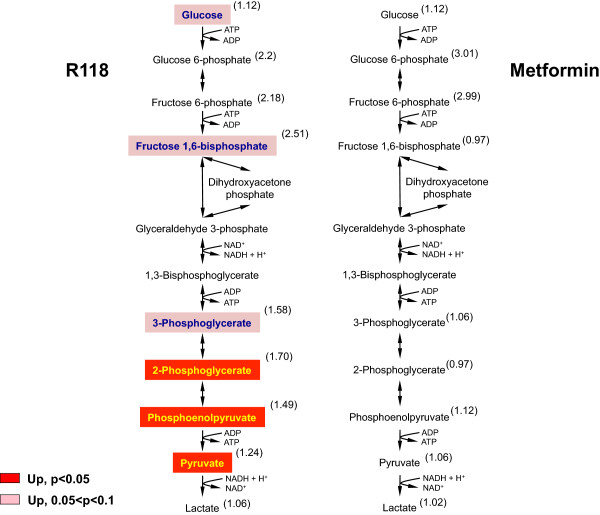
**Increased skeletal muscle glycolysis in DIO mice chronically treated with R118.** Male HFD DIO C57BL/6 mice (21 weeks old, 17 weeks on HFD) were fed using control HFD, HFD formulated with 200 mg/kg R118, or HFD formulated with 5 g/kg metformin (n = 12/group). After five weeks of treatment, liver, skeletal muscle, adipose tissue, and plasma were collected for half of the mice in each group. For the remaining mice, compound-formulated HFD was replaced with control HFD for 24 hours (washout), followed by similar sample collection. Detected changes in skeletal muscle glycolytic intermediates for the drug washout samples are shown mapped onto the glycolysis pathway. Numbers in parentheses indicate relative fold change for drug treatment compared to control HFD. Biochemical data were analyzed using Welch’s two-sample t-tests.

Figure 2 compares the effect of both R118 and metformin treatment on glycolytic intermediates in skeletal muscle measured after compound washout. R118-treated muscles displayed significant elevations in intermediates produced during the terminal steps of glycolysis, along with trending increases in intermediates found in the initial enzymatic steps. In addition, pyruvate levels were elevated without changes in lactate. Taken together, these results support an R118-mediated increase in glucose uptake and eventual disposal via glycolysis and tricarboxylic acid cycle (TCA) oxidation. In contrast to R118, metformin treatment resulted in non-significant elevations in early glycolytic intermediates with no discernible changes over control HFD treatment downstream of fructose-6-phosphate, suggesting that disposal of glucose in metformin-treated skeletal muscle occurs via non-oxidative pathways.

**Figure 3:**
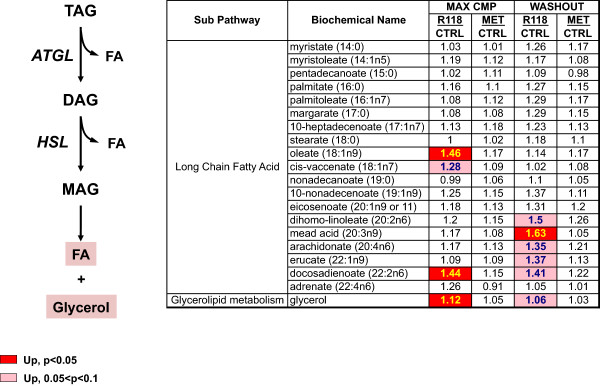
**Increased skeletal muscle lipolysis in DIO mice chronically treated with R118.** Male HFD DIO C57BL/6 mice (21 weeks old, 17 weeks on HFD) were fed using control HFD, HFD formulated with 200 mg/kg R118, or HFD formulated with 5 g/kg metformin (n = 12/group). After five weeks of treatment, liver, skeletal muscle, adipose tissue, and plasma were collected for half of the mice in each group (max cmp). For the remaining mice, compound-formulated HFD was replaced with control HFD for 24 hours (washout), followed by similar sample collection. Heatmap of detected changes in skeletal muscle long chain fatty acids and glycerol for both R118 and metformin-treated mice shown relative to control HFD is presented alongside a simple scheme illustrating the triacylglycerol lipolysis pathway. TAG = triacylglycerol, DAG = diacylglycerol, MAG = monoacylglycerol, FA = fatty acid, ATGL = adipose triglyceride lipase, HSL = hormone sensitive lipase. Biochemical data were analyzed using Welch’s two-sample t-tests.

Measurements of long chain fatty acids (LCFA) in skeletal muscle from R118-treated animals reveals trending elevations in some LCFA species concomitant with a trending elevation in glycerol (Figure 3). This pattern was observed both in the presence of R118 and also following R118 washout but was not apparent in skeletal muscle from metformin-treated animals either in the presence or absence of drug. Increases in free fatty acids coincident with elevated glycerol are characteristic features of triacylglycerol lipolysis, which suggests that breakdown of intramuscular lipids is increased with chronic R118 treatment.

### R118 has limited effects on hepatic fatty acid and glucose metabolism

**Figure 4:**
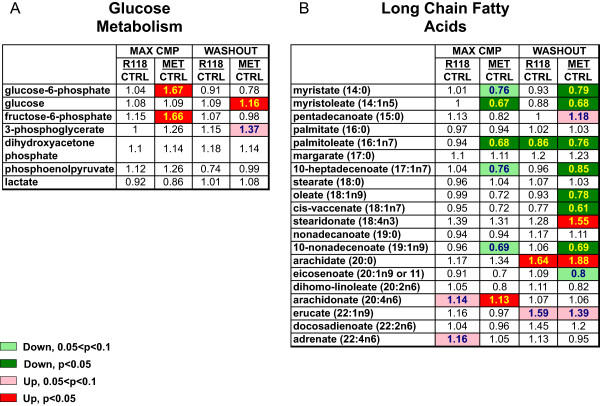
**Metformin has a more pronounced effect on hepatic glucose metabolism and long chain fatty acids compared to R118.** Male HFD DIO C57BL/6 mice (21 weeks old, 17 weeks on HFD) were fed using control HFD, HFD formulated with 200 mg/kg R118, or HFD formulated with 5 g/kg metformin (n = 12/group). After five weeks of treatment, liver, skeletal muscle, adipose tissue, and plasma were collected for half of the mice in each group (max cmp). For the remaining mice, compound-formulated HFD was replaced with control HFD for 24 hours (washout), followed by similar sample collection. Detected changes in hepatic intermediates of glucose metabolism **(Panel A)** and long chain fatty acids **(Panel B)** for both R118 and metformin-treated mice are shown relative to control HFD in heatmap format. Biochemical data were analyzed using Welch’s two-sample t-tests.

Figure 4 shows the changes relative to the HFD control in intermediates of glucose metabolism and long chain fatty acids for R118 and metformin treatment. Metformin treatment surprisingly did not dramatically impact hepatic glucose metabolism with the caveat that the samples were collected in the fed state where insulin suppression of glucose production would be maximal. Some intermediates were significantly elevated (glucose-6-phosphate, fructose-6-phosphate) although lactate was decreased (non-significant). Hepatic glucose did increase significantly when metformin was withdrawn, which would support a drug-related effect on hepatic glucose production.

Chronic metformin treatment resulted in more striking changes in levels of hepatic LCFAs, with some increasing while others decreased, possibly reflecting contributions from both mitochondrial and peroxisomal lipid metabolism [18] as well as decreased LCFA synthesis. Although R118 possesses substantially greater potency in complex I inhibition and AMPK activation *in vitro* compared to metformin (69 nM versus 27 mM on complex I inhibition), its chronic effects on hepatic glucose metabolism and LCFA levels were much less apparent than metformin.

### Increased 3-hydroxybutyrate (BHBA) is an acute response to both R118 and metformin treatment

**Figure 5:**
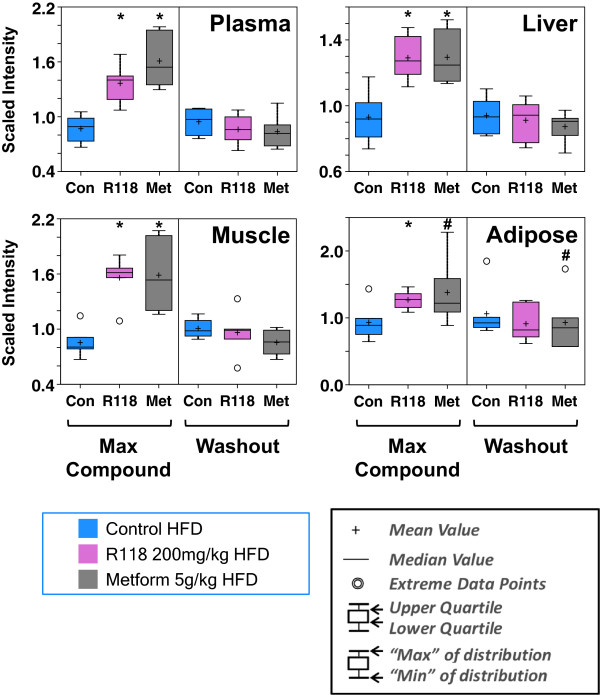
**Acute elevation in BHBA levels mediated by both R118 and metformin.** Male HFD DIO C57BL/6 mice (21 weeks old, 17 weeks on HFD) were fed using control HFD, HFD formulated with 200 mg/kg R118, or HFD formulated with 5 g/kg metformin (n = 12/group). After five weeks of treatment, liver, skeletal muscle, adipose tissue, and plasma were collected for half of the mice in each group (max compound). For the remaining mice, compound-formulated HFD was replaced with control HFD for 24 hours (washout), followed by similar sample collection. Box plots are shown of BHBA measurements from plasma, liver, skeletal muscle, and adipose tissue from R118-fed, metformin-fed, and control HFD fed animals, both in the presence of drug and after drug washout. Data are scaled such that the median value measured across all samples was set to 1.0. * represents p < 0.05; # represents 0.05 < p < 0.1.

BHBA, a ketone body that is generated from acetyl CoA derived usually from mitochondrial fat oxidation, was significantly increased with chronic treatment of DIO mice (Figure 5) across all sampled compartments with R118 and most compartments with metformin (trending in adipose tissue). In all matrices, BHBA levels were elevated only in the presence of the drugs, remaining at control HFD levels after drug washout despite changes in fatty acid metabolism becoming more evident in the washout samples for both R118 and metformin (Figures 3 and 4), suggesting that elevated BHBA is an acute response to each drug rather than a chronic adaptation.

### R118 and metformin display differential effects on purine metabolites

**Figure 6:**
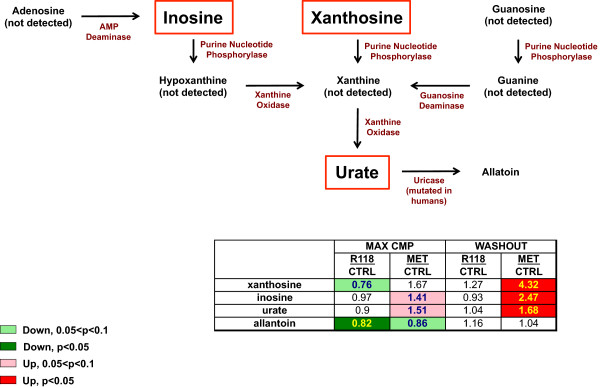
**Chronic metformin treatment results in increased plasma levels of purine metabolites.** Male HFD DIO C57BL/6 mice (21 weeks old, 17 weeks on HFD) were fed using control HFD, HFD formulated with 200 mg/kg R118, or HFD formulated with 5 g/kg metformin (n = 12/group). After five weeks of treatment, liver, skeletal muscle, adipose tissue, and plasma were collected for half of the mice in each group (max cmp). For the remaining mice, compound-formulated HFD was replaced with control HFD for 24 hours (washout), followed by similar sample collection. Detected changes in plasma purine metabolites for both R118 and metformin-treated mice are shown relative to control HFD in heatmap format, along with a schematic illustrating the purine catabolism pathway. Intermediates highlighted using red boxes are significantly elevated in metformin washout samples.

In plasma, several intermediates of purine breakdown were significantly elevated in metformin-treated but not R118-treated mice (Figure 6). The perturbations of this pathway were sustained and increased further with metformin washout. Examination of the detected purine metabolites across liver, skeletal muscle, and adipose revealed no obvious candidate tissue targeted by metformin that is primarily responsible for the systemic changes (data not shown). In a separate study, we have observed dose-dependent elevations in both plasma xanthine and uric acid from DIO mice after chronic treatment with increasing doses of metformin-formulated HFD (Additional file 3: Figure S3) whereas these metabolites were slightly decreased with R118 at 300 mg/kg HFD, suggesting that this pathway is indeed altered with metformin treatment. These metabolites were significantly increased in plasma samples from mice collected at two different time points in the active period, reinforcing the observations made in the metabolomics analysis.

## Discussion

R118 and metformin indirectly activate AMPK by inhibiting complex I of the mitochondrial respiratory chain, although R118 is more potent *in vitro* against both these molecular targets compared to metformin. The compounds display similar activities in cellular assays of glucose and fat metabolism (data not shown), albeit with significant differences in potency between metformin compared to R118. Chronic treatment with each of these compounds improves systemic glucose homeostasis in DIO mice. Despite having a similar mechanism of action, our metabolomics study presented here highlights the contrast in tissue-selective effects of the two compounds on metabolic pathways, with a more profound impact of metformin observed on liver metabolic pathways and R118 on skeletal muscle.

Plasma BHBA was elevated with both R118 and metformin treatment of DIO mice, an indirect indicator of upregulated fatty acid oxidation by both compounds. Despite the effects on LCFA species in liver and skeletal muscle becoming more marked with washout of metformin and R118 respectively, BHBA levels in all matrices returned to control levels when both drugs were removed, suggesting that BHBA elevation is an acute marker for the inhibitory activity of each drug against complex I rather than a chronic adaptation to repeated AMPK stimulation. Complex I functions to transfer electrons from nicotinamide adenine dinucleotide hydrate (NADH) to the mitochondrial respiratory chain, and in the process regenerates nicotinamide adenine dinucleotide (NAD^+^). When the drugs are present, complete TCA cycle oxidation of the acetyl CoA molecules released by increased breakdown of LCFAs is likely impaired due to the complex I block. Acetyl CoA molecules in excess of TCA cycle capacity may be converted to BHBA in a reaction that produces NAD^+^, helping to compensate for reduced complex I function. Elevated plasma BHBA levels were also observed in *db/db* leptin receptor-deficient mice orally dosed with R419, a mechanistic analog of R118, providing additional support for BHBA as a marker for complex I inhibition [4].

Repeated stimulation of AMPK, such as occurs with endurance training, induces metabolic remodeling of skeletal muscle [19–21]. These adaptations serve to increase nutrient delivery and also to increase oxidative metabolism, consequently enabling the skeletal muscle to better maintain metabolic homeostasis during periods of energetic deficit. R118 increased exercise capacity in HFD-fed mice, which was accompanied by increased activity of skeletal muscle citrate synthase, a mitochondrial enzyme [1]. In the present study, chronic R118 treatment increased skeletal muscle glucose uptake and metabolism. Additionally, the increases in the levels of downstream glycolytic intermediates along with unchanged lactate support an increase in oxidative metabolism of glucose, which would be consistent with the increased mitochondrial function suggested by the elevated citrate synthase activity.

Another adaptation that occurs in response to endurance exercise training is an increased utilization of lipids for adenosine triphosphate (ATP) production [22, 23]. Factors contributing to this increased reliance on lipid fuels include increased oxidative capacity due to greater mitochondrial density, enhanced nutrient and oxygen delivery to skeletal muscle resulting from increased capillary density, and also increased mobilization and transport of fatty acids [24], processes that are coordinately regulated. Lipid fuels can either come from plasma, which contains adipose-derived LCFAs liberated via triacylglycerol (TAG) lipolysis, or from increased lipolysis of intramuscular TAGs [25–27]. R118-treated skeletal muscle displayed concurrent elevations in several LCFAs as well as glycerol, indicative of intramuscular TAG lipolysis, both in the presence of drug and also when drug was removed (Figure 3). Although the increased exercise performance resulting from R118 treatment of aged, obese mice was mainly attributed to R118-mediated normalization of microvascular function, skeletal muscle citrate synthase activity was also increased, indicating an improvement in mitochondrial function which could also have a positive effect on exercise capacity.

The impact of metformin on skeletal muscle glucose metabolism in our study is minimal, especially when compared to R118. The direct effects of metformin on skeletal muscle insulin resistance are still debated [8, 9] although metformin treatment has been shown to increase muscle AMPK activity in human subjects [28]. In this study, metformin increased the glucose disposal rate after 10 weeks of treatment. However, this was accompanied by a small but significant decrease in mean body weight. Glucose disposal was proposed to occur mainly through nonoxidative mechanisms; no increase in glucose oxidation was detected whereas muscle glycogen content was increased. No significant effects on skeletal muscle glycogen metabolism were observed for metformin in this metabolomics study (data not shown) but the pattern in the levels of detected glycolytic intermediates is consistent with nonoxidative glucose disposal.

The overall reduction in hepatic LCFAs observed with chronic metformin treatment correlates well with the selective effects of metformin in HFD-fed wild type mice but not mice expressing alanine knock-in mutations at the AMPK phosphorylation sites in both ACC1 and ACC2 (AccDKI) [13]. In contrast to the wild type mice where metformin treatment reduced hepatic lipid content, metformin had no effects on hepatic lipid levels or hepatic insulin sensitivity in the AccDKI mice, highlighting the importance of AMPK-mediated regulation of lipid metabolism in the liver in the improved maintenance of glucose homeostasis observed with chronic metformin treatment.

One clear distinction between the two compounds was the novel finding that several intermediates of purine metabolism were elevated in plasma from metformin-treated but not R118-treated DIO mice. This elevation was further enhanced with drug washout, which suggests that this is a chronic effect of metformin dosing. We analyzed plasma from an independent metformin dose-titration study performed in DIO mice and found a dose-dependent elevation in both plasma xanthine and uric acid levels, whereas treatment using R118 at 300 mg/kg in HFD displayed the opposite effect (Additional file 3: Figure S3). This elevation was detected in plasma samples harvested at two different time points in the active cycle, providing evidence that clearly corroborates the results from the *in vivo* metabolomics study. These data suggest that metformin treatment may perturb carbon flow through purine metabolic pathways, an effect that is specific to metformin since R118 treatment produced opposite results. One alternative mechanism proposed for indirect activation of AMPK by metformin is via a direct effect on adenosine deaminase, an enzyme that catalyzes conversion of adenosine to inosine [29]. One might then expect a resulting decrease in metabolite concentrations downstream of adenosine, the opposite of what was observed in this metabolomics study. Of interest, it was recently reported that metformin treatment of breast cancer cells resulted in a significant accumulation of 5-formimino-tetrahydrofolate, a carrier of activated one-carbon units that contributes to de novo purine synthesis [30], a finding which may be related to the increase in purine catabolism observed here.

## Conclusions

In conclusion, our study clearly demonstrates distinct tissue-selective effects of R118 and metformin on nutrient metabolism, despite the characterization of both compounds as complex I inhibitors that display similar cellular and pharmacological activities. R118 elicited more profound changes in glucose and lipid metabolism than metformin in skeletal muscle, changes that may have contributed to the enhancements in exercise capacity reported for R118 in aged, obese mice and if translatable to humans, may be beneficial in the treatment of muscle complications resulting from metabolic dysfunction.

## Electronic supplementary material

Additional file 1: Figure S1: Improvement of glucose tolerance by both R118 and metformin in DIO mice used for the metabolomics analysis. Male HFD DIO C57BL/6 mice (21 weeks old, 17 weeks on HFD) were fed using control HFD, HFD formulated with 200 mg/kg R18, or HFD formulated with 5 g/kg metformin (n = 12/group). ***A:*** OGTT and glucose AUC (area under the curve) following 4-week treatment. GraphPad Prism version 6.0 was used for 2-way (OGTT) and 1-way (glucose AUC) ANOVA analysis of data as well as calculation of glucose AUC. Asterisks *, **, and **** represent p < 0.05, p < 0.01, and p < 0.0001, respectively. ***B:*** Body weight gain during 5-week treatment period. (PDF 288 KB)

Additional file 2: Figure S2: Plasma drug levels in samples harvested in the presence of drug and 24 hours after drug washout. Male HFD DIO C57BL/6 mice (21 weeks old, 17 weeks on HFD) were fed using control HFD, HFD formulated with 200 mg/kg R18, or HFD formulated with 5 g/kg metformin (n = 12/group). After five weeks of treatment, plasma samples were collected as in Figure 1 and analyzed by LC/MS/MS to quantitatively determine R118 and metformin concentration values. BLOQ: below limit of quantitation. (PDF 218 KB)

Additional file 3: Figure S3: Serum xanthine and uric acid levels in DIO mice dosed for 6 weeks using either R118 300 mg/kg HFD or increasing doses of metformin (2.5 g/kg, 5 g/kg, 7.5 g/kg, and 10 g/kg HFD) (n = 12/group). Blood was collected from three mice/group at 4 different timepoints: 6 AM, 10 AM, 4 PM, and 9 PM. Data are shown for the 6 AM and 9 PM timepoints, when mice are still in their active cycle. Serum xanthine and uric acid levels were measured using commercially available kits (Life Technologies). Data was analyzed in GraphPad Prism version 6.0 using 1-way ANOVA with Dunnett’s test against the HFD control. Asterisks *, ** and **** represent p < 0.05, p < 0.01, and p < 0.0001, respectively. (PDF 273 KB)

## Abbreviations

AMPK: 5′-adenosine monophosphate-activated protein kinase
ACC: Acetyl-CoA carboxylase
DIO: Diet-induced obesity
HFD: High fat diet
OGTT: Oral glucose tolerance test
TCA: Tricarboxylic acid cycle
LCFA: Long chain fatty acid
BHBA: 3′-hydroxybutyrate
NADH: Nicotinamide adenine dinucleotide hydrate
NAD+: Nicotinamide adenine dinucleotide
ATP: Adenosine triphosphate
TAG: Triacylglyceride.
