# Supplementary figures and images for: Global metabolite profiling of mice with high-fat diet-induced obesity chronically treated with AMPK activators R118 or metformin reveals tissue-selective alterations in metabolic pathways
Source: BMC Res Notes. 2014 Sep 25;7:674. doi: 10.1186/1756-0500-7-674 (PMC4182811; doi:10.1186/1756-0500-7-674)

Figure S1

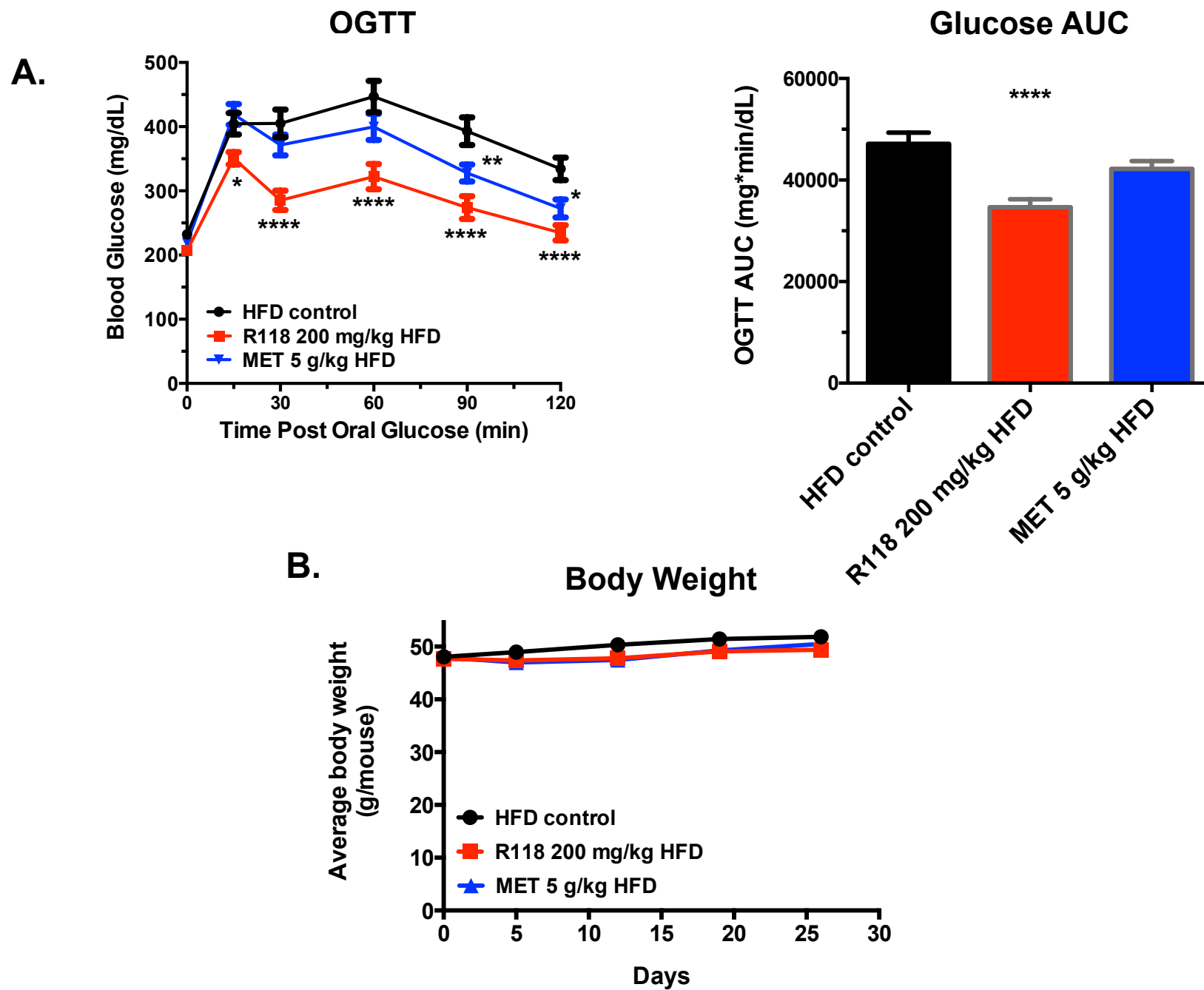

Supplement: Supplementary file 1 — Additional file 1: Figure S1: Improvement of glucose tolerance by both R118 and metformin in DIO mice used for the metabolomics analysis. Male HFD DIO C57BL/6 mice (21 weeks old, 17 weeks on HFD) were fed using control HFD, HFD formulated with 200 mg/kg R18, or HFD formulated with 5 g/kg metformin (n = 12/group). A: OGTT and glucose AUC (area under the curve) following 4-week treatment. GraphPad Prism version 6.0 was used for 2-way (OGTT) and 1-way (glucose AUC) ANOVA analysis of data as well as calculation of glucose AUC. Asterisks *, **, and **** represent p < 0.05, p < 0.01, and p < 0.0001, respectively. B: Body weight gain during 5-week treatment period. (PDF 288 KB) [file 13104_2014_3200_MOESM1_ESM.pdf]

Figure S2

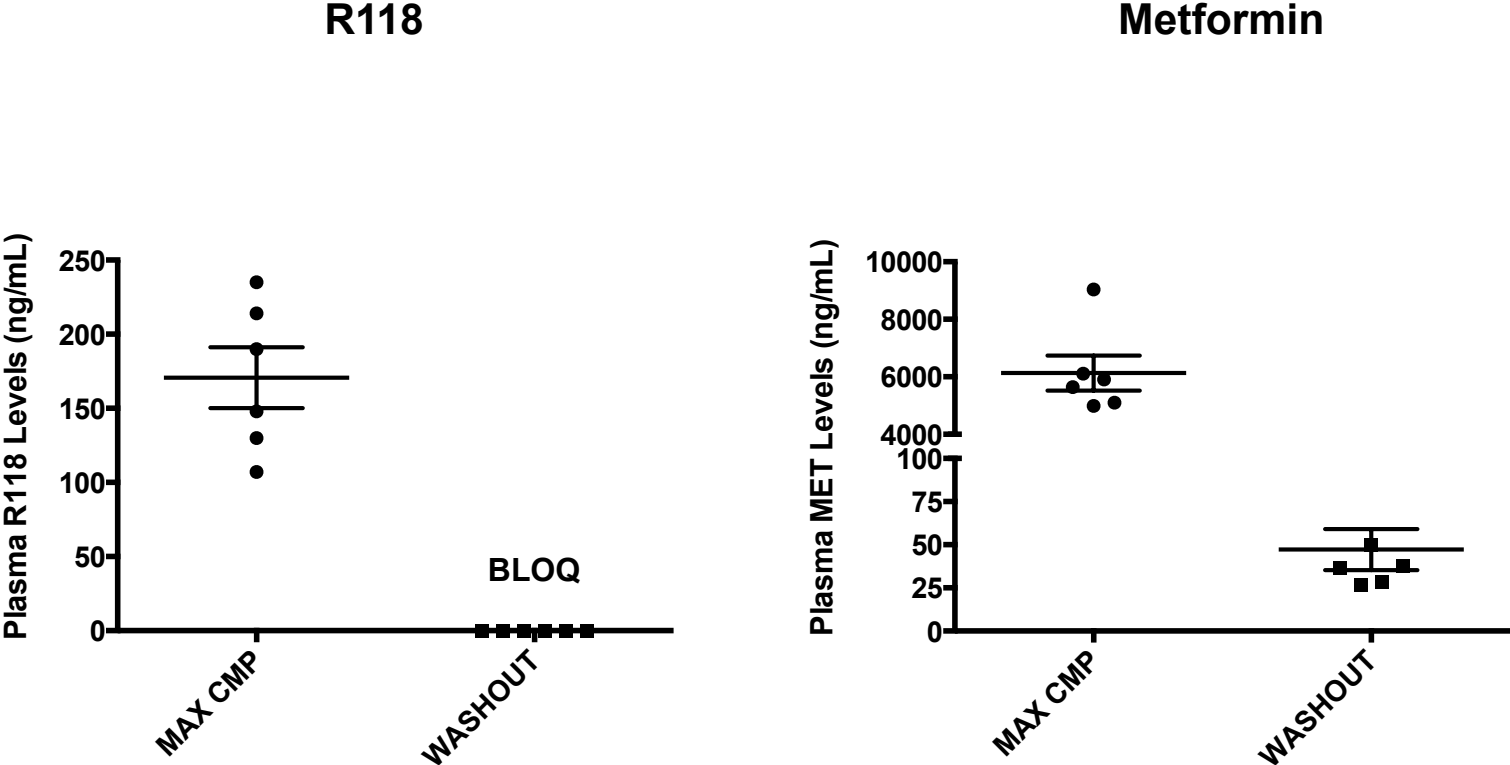

Supplement: Supplementary file 2 — Additional file 2: Figure S2: Plasma drug levels in samples harvested in the presence of drug and 24 hours after drug washout. Male HFD DIO C57BL/6 mice (21 weeks old, 17 weeks on HFD) were fed using control HFD, HFD formulated with 200 mg/kg R18, or HFD formulated with 5 g/kg metformin (n = 12/group). After five weeks of treatment, plasma samples were collected as in Figure 1 and analyzed by LC/MS/MS to quantitatively determine R118 and metformin concentration values. BLOQ: below limit of quantitation. (PDF 218 KB) [file 13104_2014_3200_MOESM2_ESM.pdf]

Figure S3

Xanthine

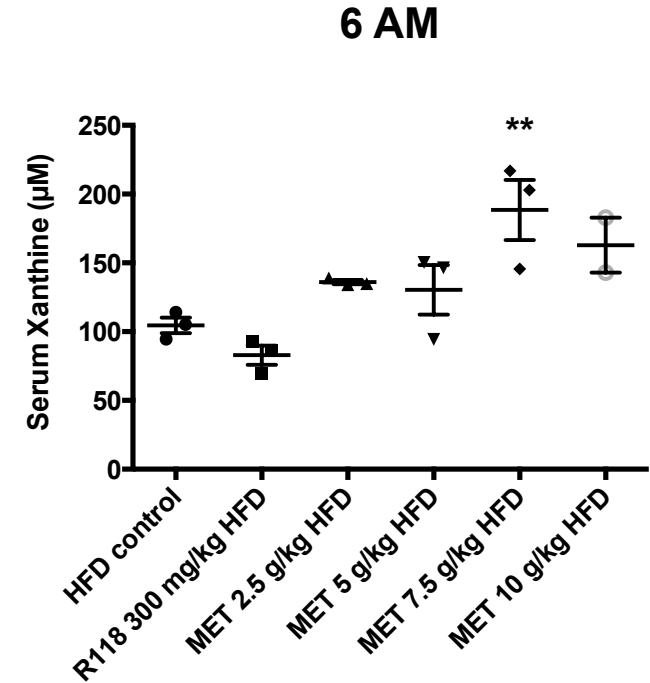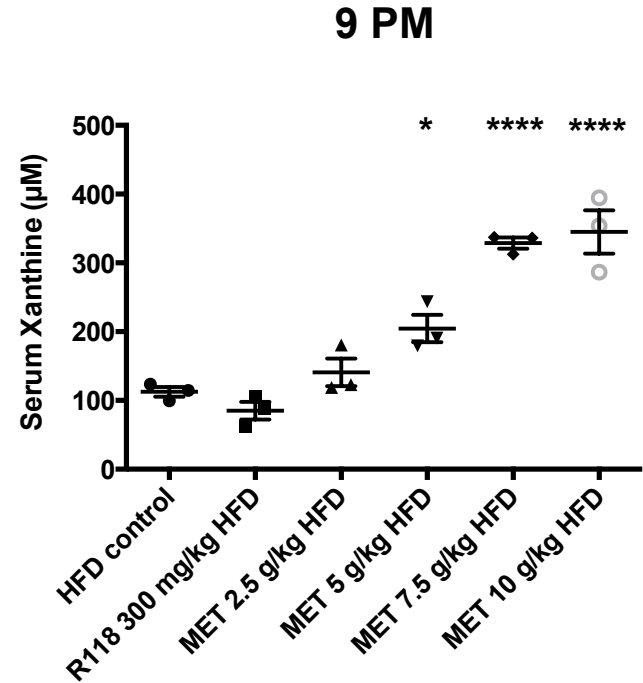

Uric Acid

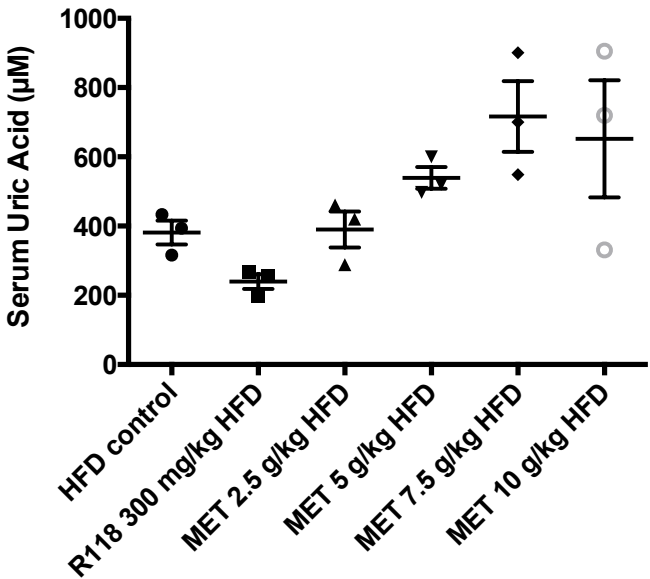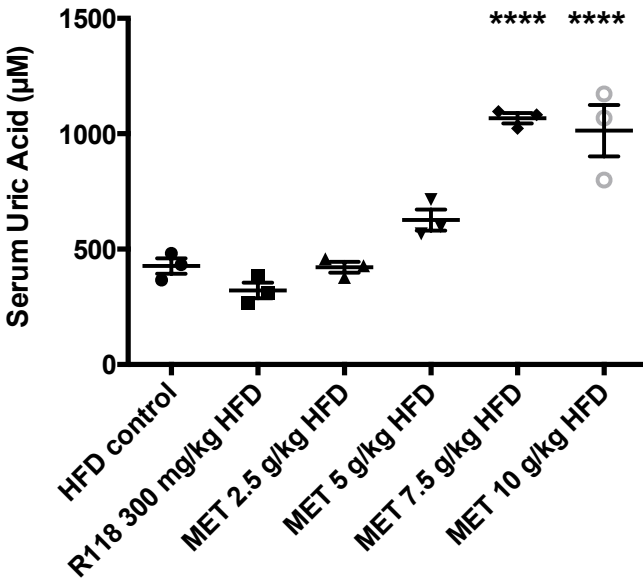

Supplement: Supplementary file 3 — Additional file 3: Figure S3: Serum xanthine and uric acid levels in DIO mice dosed for 6 weeks using either R118 300 mg/kg HFD or increasing doses of metformin (2.5 g/kg, 5 g/kg, 7.5 g/kg, and 10 g/kg HFD) (n = 12/group). Blood was collected from three mice/group at 4 different timepoints: 6 AM, 10 AM, 4 PM, and 9 PM. Data are shown for the 6 AM and 9 PM timepoints, when mice are still in their active cycle. Serum xanthine and uric acid levels were measured using commercially available kits (Life Technologies). Data was analyzed in GraphPad Prism version 6.0 using 1-way ANOVA with Dunnett’s test against the HFD control. Asterisks *, ** and **** represent p < 0.05, p < 0.01, and p < 0.0001, respectively. (PDF 273 KB) [file 13104_2014_3200_MOESM3_ESM.pdf]
